# Investigation of causes of ceiling effects on working alliance measures

**DOI:** 10.3389/fpsyg.2022.949326

**Published:** 2022-07-22

**Authors:** Scott T. Meier

**Affiliations:** Department of Counseling, School and Educational Psychology, The State University of New York at Buffalo, Buffalo, NY, United States

**Keywords:** psychotherapy, working alliance, measurement, replication, ceiling effects, item response

## Abstract

The presence of ceiling effects on measures of working alliance is important because they (a) may moderate the observed size of the alliance-outcome correlation and (b) have implications for how quickly the alliance is formed and when. Despite this, little is known about ceiling effects on alliance measures, particularly about potential causes. This study attempted to replicate findings of ceiling effects using a 7-item version of the Working Alliance Inventory (WAI) (Horvath and Greenberg, 1989) accessed in an archival database of 616 parolees enrolled in a drug abuse treatment study. Item response patterns on alliance and related measures were examined to explore potential methodological and theoretical factors that could produce ceiling effects. Analyses revealed ceiling effects on alliance measures assessing relationships with counselors and parole officers as well as floor effects (indicating highly positive appraisals) in measures of outcome expectations with counselors and parole officers. No ceiling effects were found with measures of drug use problems or negative affect. Item responses on the alliance and outcome expectations measures evidenced high consistency where many respondents endorsed the same choice on the 5-point response format across all items on the scale. Ceiling effects offer a potential marker of the working alliance at the scale level, while consistent response choice may provide a specific behavioral marker at the item level. Discussion focuses on theoretical implications and directions for future research in psychotherapy.

## Introduction

Counseling and psychotherapy researchers have found two important results related to the working alliance between client and therapist. First, researchers using meta-analysis have established an overall effect size of approximately 0.30 between scores on working alliance and outcome measures (Horvath and Symonds, 1991; Martin et al., 2000; Horvath and Bedi, 2002; Del Re et al., 2012; Flückiger et al., 2012, 2018). A reasonable interpretation of these consistent findings is that a correlational and possible causal relation exists between alliance and psychotherapy outcome. Second, client-completed alliance measures frequently evidence a ceiling effect early in therapy, suggesting that many clients quickly develop a strong affiliation with their therapist (Tryon et al., 2007; Hall et al., 2012; Reese et al., 2013; Babatunde et al., 2017; Paap et al., 2019; Meier and Feeley, 2021). Ceiling effects refer to a set of scores clustering toward the top of the range for an item, subscale, or total scale score.

Because traditional test development procedures attempt to identify and eliminate items whose score distributions are skewed, ceiling effects are relatively uncommon in psychological tests. The use of Likert response formats with more than 2 response options typically leads to item scores with higher reliability estimates, item-total correlations, and factor loadings (Maul, 2017) as well as fewer ceiling or floor effects. Developers of alliance measures, including the frequently employed Working Alliance Inventory (WAI; Horvath and Greenberg, 1989), followed traditional test construction procedures designed to maximize variability in item response and subsequent aggregated scale scores (Meier and Feeley, 2021). Given the procedures employed in creating alliance measures, it remains unclear why ceiling effects are commonly observed on client-rated alliance measures.

Some researchers favor measurement problems as the chief explanation for ceiling effects on alliance scales (Paap et al., 2019; Baldwin and Goldberg, 2021). Describing the effects of restricted range on alliance-outcome correlations, Baldwin and Goldberg (2021, p. 28) wrote that “we believe that alliance measures may not be sufficiently sensitive to discriminate variability in the alliance.” Paap et al. (2019) also favored a technical explanation, suggesting that ceiling effects result from a failure to construct a comprehensive response format for test items as well as client characteristics such as socially desirable responding.

On the other hand, ceiling effects on client-rated alliance measures may be a consequence of theoretical factors related to the development of the therapeutic relationship (Meier and Feeley, 2021). Using data from two previously published meta-analyses, Meier and Feeley produced 92 estimates of ceiling effects based on 37 studies with 6,439 participants. They found moderate to large ceiling effects across multiple measures of client-rated alliance (e.g., the WAI and SRS) as well as time of administration. While the working alliance has typically been defined in terms of theoretical content such as tasks, goals, and bond (Bordin, 1976, 1994), Meier and Feeley concluded that for many clients, alliance development is not gradual or incremental, but occurs abruptly, non-linearly, and early in therapy. Meier and Feeley suggested that a key element is a threshold structure where clients shift to an experience of the therapeutic relationship as *established*.

Given that many clients may wish to offer positive feedback to their therapist on alliance measures, social desirability, where individuals provide desired responses in relation to the assessment purpose, is an individual differences variable of interest. Research conducted by Reese et al. (2013) and Sturgiss et al. (2019), however, failed to find significant correlations between measures of social desirability and alliance. In contrast, Osborne and Blanchard (2011) studied the effect of random responding on outcome measures used to evaluate an educational intervention. They created a simple random responding scale with items that should be answered in a particular direction by 0 or 100% of the respondents. Deviations from the expected response allowed identification of random responders, who presumably lacked motivation to provide valid responses. Osborne and Blanchard found that random responders evidenced (a) lower scores on study tests and (b) failed to improve on scores intended to assess change from pre- to post-test after an educational intervention intended to improve student learning and retention. Non-random responders had higher scores and did show growth on pre-post scores.

This study sought to replicate ceiling effect findings on alliance measures using a diverse sample and methodological conditions. To extend previous findings with ceiling effects, an archival dataset was sought for this study with a sample different from that typically employed in alliance-outcome studies; Meier and Feeley (2021) noted that one of the limitations of their review was that study samples consisted primarily of female, younger, and Caucasian clients. In addition, the archival data will be explored for patterns of item responses that might reflect one or more individual differences variables that could potentially explain ceiling effects on alliance scores (Osborne and Blanchard, 2011; Reese et al., 2013; Sturgiss et al., 2019).

## Methodology

### Procedure

An archival database with working alliance data was located at the University of Michigan Inter-University Consortium for Political and Social Research.^1^ The dataset was collected for a large-scale drug abuse treatment study called Step ’N Out where participants were randomly assigned to a behavioral management treatment group or a control group (Friedmann, 2011). Data collection occurred from 2002 to 2006 in parole offices in six United States cities; a 2015 version of the dataset was available at the website and employed in this study. Participants were 18 years of age or older, English speaking, evidenced drug dependence related to the most recent incarceration, and had moderate to high risk for recidivism or relapse.

### Participants

The full database of Step’N Out participants at baseline included 616 individuals whose mean age was 33.70 (SD = 9.04, range 18–61). The sample consisted of 83% males and 17% females; 55% were Black, 33% Caucasian, 10% Other, and 2% Native American individuals. Completed school grade ranged from 5 to 18, with most individuals attending some level of high school [i.e., grades 10 (19%), 11 (20%), or 12 (34%)]. Sample size varied by measure and time of administration (see Table 1). These participants were of interest because (a) compared to traditional psychotherapy clients, mandated clients may be less likely to develop a working alliance and engage in counseling (cf. Henskens et al., 2018; Sturm et al., 2019), and (b) they represent a more diverse sample than those previously studied in alliance research (cf. Meier and Feeley, 2021).

**TABLE 1:** Descriptive statistics, coefficient alpha, and pearson correlations.

Scale	*n*	*M* (*SD*)	Range	Alpha	2	3	4	5	6	7	8
1. WA-CO	316	27.12 (5.96)	5–35	0.95	0.60**	0.08	−0.05	−0.63**	−0.50**	0.16**	−0.02
2. WA-PO	316	26.89 (6.20)	5–35	0.95		0.10	−0.05	−0.53**	−0.72**	0.19**	0.01
3. DUPS	464	45.10 (11.86)	13–65	0.93			0.25**	−0.15**	−0.10	0.35**	0.07
4. NAS	472	21.50 (5.99)	40	0.81				0.01	0.04	−0.06	−0.08
5. CO-ACTS	359	10.46 (3.72)	5–25	0.92					0.63	−0.13*	−0.02
6. PO-ACTS	369	10.42 (4.18)	5–25	0.88						−0.12*	−0.09
7. Age	642	33.62 (8.95)	18–61								0.14**
8. Grade	643	11.14 (1.88)	5–18								

**p < 0.01.

*p < 0.05.

WA-CO is the working alliance scale, counselor version; WA-PO, working alliance scale, parole officer version; DUPS, drug use problems scale; NAS, negative affect scale; CO-ACTS, counselor–activities scale; PO-ACTS, parole officer–activities scale. DUPS and NAS data were collected at baseline; WA-CO, WA-PO, CO-ACTS, and PO-ACTs were administered at 3-month followup. Respondents who failed to complete all items on a particular measure were not included in the analyses.

### Measures

The Step’N Out study was conducted *via* three assessment waves (baseline, 3 months, 9 months). Multiple unpublished and several published scales and items, utilizing self-report and interview questions, were employed to collect data about working alliance, parole activities and violations, sociodemographic background, family and peer relations, health and psychological status, drug use, criminal behavior, drug use history, and HIV/AIDS risk behaviors (Friedmann et al., 2008). In addition to the working alliance measures (administered at the 3 month wave), items were chosen for this study that had been administered at baseline and 3 months, contained psychosocial content similar to and different from the alliance measures, and employed 5-point Likert response formats that could be assembled into scales with acceptable estimates of internal consistency. These criteria led to creation of four scales assessing self-reported perceptions of drug-related problems, negative affect, and expectations regarding activities respondents performed with counselors and parole officers.

#### Working alliance inventory–short form

The 7-item, 5-point self-report Likert scale WAI (Horvath and Greenberg, 1989) was employed to obtain two alliance scores relevant to respondents’ counselor and parole officer. These versions are referred to as WAI-CO and WAI-PO; “counselor” was the focus of items on the WAI-CO and “parole officer” on the WAI-PO. Sample items include “My work with my counselor is important to me” and “My work with my parole officer is important to me.” Both versions of the WAI scales were administered 2–3 months post-baseline; high scores indicate a stronger alliance. As shown in Table 1, coefficient alpha equaled 0.95 for both the WAI-CO and WAI-PO. Walters (2016) reported that scores averaged between both WAI measures predicted respondents’ subsequent drug use, arrests, and days in jail in the next wave of data collection (9 months post-baseline). Higher WAI scores were associated with lower drug, arrest, and jail outcomes.

#### Parole officer–expectations scale working alliance scale, parole officer

Collected at 3-month followup, this self-report 5-point Likert scale focused on outcome expectations, that is, knowledge and behaviors the respondent developed with a parole officer to succeed at parole. Originally 6 items, one item was dropped for this study because of a low item-total correlation (0.02). Sample items include “My parole officer explained exactly what I have to do to succeed on parole” and “My parole officer and I made a contract about the things I should and should not do while on parole.” Higher scores on the EXP-PO indicate a lower level of activities. The 5-item scale had a coefficient alpha of 0.88.

#### Counselor–expectations scale working alliance scale, counselor

Parallel to the EXP-PO, this scale was collected at 3-month followup and focused on outcome expectations developed with the treatment counselor. A self-report 5-point Likert scale, one item was dropped from the original 6-item scale because of a low item-total correlation (0.00). Sample items include “My treatment counselor explained exactly what I have to do to succeed in treatment” and “My treatment counselor and I made a contract about the things I should and should not do during treatment.” Higher scores on the EXP-CO indicate a lower level of activities. The coefficient alpha for the 5-item scale equaled 0.92.

#### Drug use problems scale

This self-report 5-point Likert scale was created by identifying item content related to self-perceived problems about drug use embedded in the Step’N Out Intake data at baseline. Data for 14 items were collected, but one item was dropped as a result of low item-total correlation (0.14). Sample items include “Your drug use is a problem for you” and “You need help in dealing with your drug use.” Higher scores indicate more concern about problems associated with drug use. Coefficient alpha equaled 0.93 for the 13-item measure.

#### Negative affect scale

Collected at baseline along with the DUPS items, this self-report 5-point Likert scale was composed of items with negative affect content. Sample items include “You have a hot temper” and “You feel sad or depressed.” Higher scores indicate stronger negative affect. Coefficient alpha equaled 0.81 for the 8-item measure.

## Results

Table 1 displays descriptive statistics, scale score range, coefficient alpha, and intercorrelations among all measures. Coefficient alpha for all scales exceeds 0.80, indicating acceptable internal consistency. Convergent validity for the alliance and expectations measures, respectively, is supported by the 0.60 correlation between WA-CO and WA-PO scores and the 0.63 correlation between EXP-CO and EXP-PO scores. Correlations between the alliance and expectations ratings for parole officers (*r* = −72) and for counselors (*r* = −0.63), however, exceed the correlation between alliance ratings of parole officers and counselors (*r* = 0.60) or expectations ratings of parole officers and counselors (*r* = 0.63). This suggests that the scale target (i.e., parole officer or counselor) influences scores more than alliance or expectations content *per se*.

Correlations in Table 1 indicate that increasing age (but not grade) is positively associated with alliance and expectations ratings for counselors and parole officers. The DUPS has the highest correlation with age, such that older respondents report more concerns with drug problems. NAS scores evidence no statistically significant correlations except with DUPS, indicating that more intense negative affect is associated with an assessment of greater drug problems. SAS’s General Linear Models procedure, useful with unequal sample sizes, was employed to compare gender and race (i.e., Black and Caucasian participants, the largest racial groups), the independent variables, on the six scale scores, the dependent variables. Participants with missing data were not included in the statistical analyses. For gender, no statistically significant results were found. Regarding race, statistically significant results were found for DUPS [*F*_(1,415)_ = 31.81, *p* < 0.001] and NAS [*F*_(1, 419)_ = 5.35, *p* < 0.03], but not for WAI-CO, WAI-PO, EXP-CO, or EXP-PO. Scores for Caucasian respondents were higher for DUPS and NA.

### Ceiling effects

In a normal distribution, the mean is positioned approximately 3 SDs from the top or bottom of the set of test scores (Meier and Feeley, 2021). If the sum of the mean and one to two SDs equals or exceeds the maximum test score, a ceiling effect is present, indicating that a substantial number of scores cluster near the top of the scale. Ceiling effects in this study were calculated by comparing the location of the scale mean to the highest possible score on the scale in SD units (Meier and Feeley, 2021). For both WA-CO and WA-PO, the highest possible score equaled 35 (7 items with a 5-point response format). The ceiling was 65 for the 13-item DU scale and 40 for the 8-item NAS. For both the EXP-CO and EXP-PO, the floor was 5, indicating positive appraisals.

Both WA-CO and WA-PO scores evidence ceiling effects (indicating positive appraisals) since their means (27.12 and 26.89, respectively) are 1.32 and 1.31 SD units from the highest possible scale score. The EXP-CO and EXP-PO both evidence floor effects (also indicating positive appraisals), as the EXP-CO mean (10.46) lays 1.47 SD units above the scale floor, while the EXP-PO mean (10.42) is 1.30 SD units above the scale floor. The DUPS mean was approximately 2 SDs below the ceiling and the NAS mean was 3 SDs below its highest score.

### Frequency effects

**FIGURE 1:**
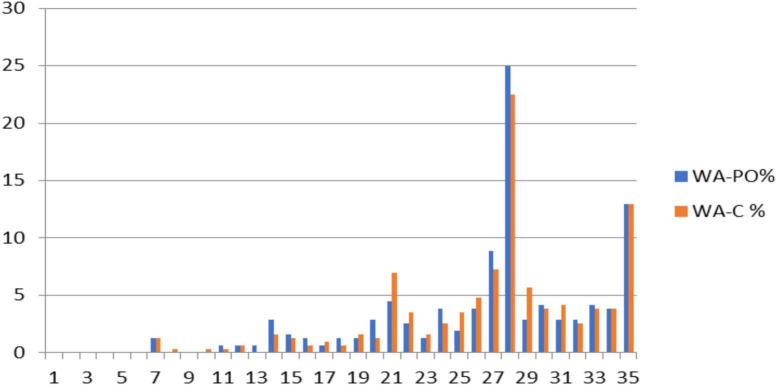
Percentage of endorsement for total scores for WA-CO and WA-PO. Nodes at 28 and 35 for total scores indicate a high proportion of respondents chose response options “4” or “5” for WA-CO and WA-PO. WA-PO refers to the parole officer version of the WAI, while WA-C refers to the counselor version.

Examination of total scores for both WA scales reveals nodes of high endorsement located at multiples of 7 (i.e., 7, 14, 21, 28, 35). Figure 1 displays the percentage of responses per item for both WA-CO and WA-PO scales. The highest percentages of endorsement occurred for total scores equaling 28 (25 and 22% for WA-CO and WA-PO, respectively) and 35 (13% for both scales). This finding indicates that endorsement of the same response option across all items happens more frequently for response options 4 and 5 than for response options 1, 2, and 3. Thus, ceiling effects on the WA scales were substantially influenced by endorsement of options 4 and 5 on those 2 items.

**FIGURE 2:**
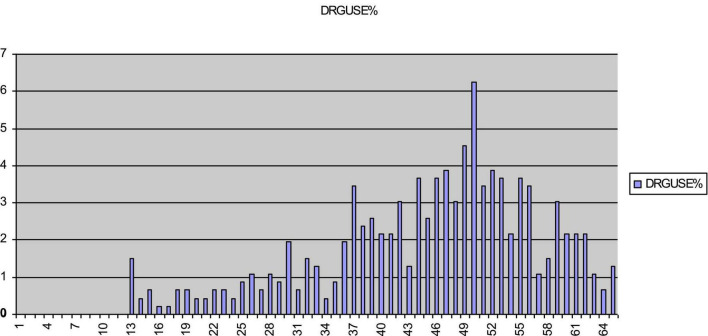
Percentage of endorsement for total scores for DUPS. Total scores for DUPS approach a normal distribution. DUPS is the drug use problems scale.

**FIGURE 3:**
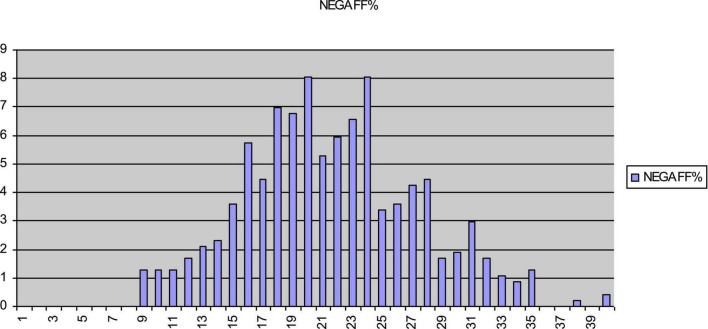
Percentage of endorsement for total scores for NAS. Total scores for NAS approach a normal distribution. NAS is the negative affect scale.

**FIGURE 4:**
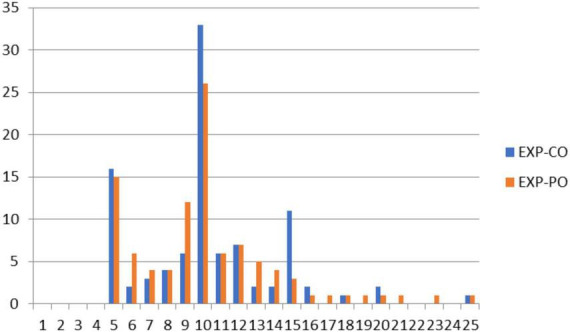
Percentage of endorsement for total scores for EXP-CO and EXP-PO. Nodes at 5 and 10 for total scores indicate a high proportion of respondents chose response options “1” or “2” for EXP-CO and EXP-PO. EXP-CO is the counselor–expectations scale, while EXP-PO refers to the parole officer–expectations scale.

Figure 2 displays scores on the DUPS that evidence a slight ceiling effect. In contrast, Figure 3 indicates that the distribution of scores for the NAS approaches a normal distribution, with a slight skew toward the floor of the scale. Of particular note, neither the DUPS nor the NAS displays the nodes apparent with both WA scales. This finding indicates that the consistent responding on WA scales did not appear on the DUPS or NAS. Figure 4 displays frequency data for total scores for the EXP-CO and EXP-PO scales. Highest percentages of endorsement occurred for total scores of 5 and 10; total percentage of endorsement equaled 49% for EXP-CO and 41% for EXP-PO. Because these scales were reversed scored, these nodes indicate very positive expectations for counselors and parole officers. Thus, scores on alliance and expectations measures evidence nodes of frequent responding that scores on drug use perceptions and negative affect do not.

### Consistency effects

For the WA measures, the frequency data indicated that many respondents endorsed only a single response across all items. To examine this tendency toward response consistency, individuals whose total scores resulted from endorsement of only a single option on the item response format were coded as “1.” All other total scores were coded as “0.” For WA-CO, 44% of all scores (*n* = 140) had a consistency score of 1; 56% (*n* = 176) had a score of 0. For WA-PO, consistent responses were found for 47% of respondents (*n* = 147) and varied responses for 53% (*n* = 169).

Table 2 displays data examining response consistency between WA scales. While 30% of respondents provided a consistent response across both WA scales, another 30% were consistent responders on only one of the WA scales. Forty percent varied their responses on both scales. Table 3 examines consistency of responses between DUPS and NAS scores. In contrast to the WA scales, only 10% of DUPS respondents (*n* = 48) and 17% of NAS respondents (*n* = 80) had a consistency score of 1. Only 2% provided consistent responses across both scales, while 23% of all responders had consistent responses to only one of these scales. In Table 4, 36% of respondents had a consistency score of 1 for both EXP-PO and EXP-CO. Thirty one percent of respondents had consistency scores of 1 for either the EXP-PO or the EXP-CO.

**TABLE 2:** Response consistency between WA-CO and WA-PO total scores.

		WA-PO		
		
		1 (Consistent)	0 (Varied)	Total
WA-CO	1 (Consistent)	30% (96)	14% (44)	44% (169)
	0 (Varied)	16% (51)	40% (125)	56% (147)
	Total	46% (147)	54% (169)	100% (316)

WA-CO refers to the working alliance-counselor scale, while WA-PO is the working alliance-parole officer scale. Numbers in parentheses are sample sizes per cell.

**TABLE 3:** Response consistency between DUPS and NAS total scores.

		DUPS		
		
		1 (Consistent)	0 (Varied)	Total
NAS	1 (Consistent)	2% (11)	15% (69)	17% (80)
	0 (Varied)	8% (36)	75% (347)	83% (383)
	Total	10% (47)	90% (416)	100% (463)

DUPS refers to the drug use problems scale, while NAS is the negative affect scale. Numbers in parentheses are sample sizes per cell.

**TABLE 4:** Response consistency between EXP-CO and EXP-PO total scores.

		EXP-PO		
		
		1 (Consistent)	0 (Varied)	Total
EXP-CO	1 (Consistent)	36% (123)	25% (84)	61% (207)
	0 (Varied)	6% (21)	33% (112)	39% (133)
	Total	42% (144)	58% (196)	100% (340)

EXP-CO refers to the expectations-counselor scale, while EXP-PO is the expectations-parole officer scale. Numbers in parentheses are sample sizes per cell.

## Discussion

This study replicated previous research that found evidence for ceiling effects on measures of working alliance where scores clustered near the top (positive) of the scale. Mean scores of the alliance with both counselors and parole officers were close to 1 SD unit near the top of the respective scales. In contrast, a normal distribution was apparent for scores on a measure of drug use problems, while scores on a negative affect scale suggested a slight floor effect. This positive clustering of scores on alliance measures resulted from ratings made by mandated, not self-referred, clients. This replication is strengthened by the finding of a floor effect on a reversed score expectations measure, indicating that positive evaluations of counselors and parole officers were not simply a result of the valence of item wording and an acquiescence bias.

Investigation of individual differences in item responding found evidence for response consistency where respondents endorsed the same response from the 5-point response format across all items on the scale. On the WA-CO and WA-PO, for example, 44 and 46% of all respondents, respectively, endorsed the same response across the 7-item scales. If this consistent responding resulted from respondents’ lack of motivation, this item response behavior should have been spread equally across response options 1, 2, 3, 4, and 5. The nodes of consistent responding, however, occurred more frequently on response options toward the positive end of the scale for WA-CO, WA-PO, EXP-CO, and EXP-PO. That is, consistent responding occurred in such a manner that provided high appraisals, reflected in total scores, for counselors and parole officers.

Individuals who responded consistently to items across scales comprised 30% of WA-CO/WA-PO scores, 2% of DUPS/NAS scores, and 36% of EXP-CO and EXP-PO scores. Thus, consistent responding appeared more frequently on scales rating counselors and parole officers, but less so on the drug use and negative affect scales. This finding suggests that consistent responding is not a result of an individual differences variable, but depends upon the specific scale. Additionally, correlations between alliance and expectations ratings for parole officers and counselors exceeded the correlation between alliance ratings for parole officers and counselors, indicating that the scale *target* (i.e., parole officer or counselor) influenced scores more than alliance or expectations *content*.

While this study’s results argue against respondents as unmotivated or random responders, social desirability remains a possible, but less likely explanation. Some respondents may have had doubts about the confidentiality of their responses, for example, believing that their scores may influence the parole process. If social desirability is an individual differences variable reflected by consistent responding, it should have been present across all scales. Consistent responding was absent, however, for DUPS and NAS. Study findings are more compatible with a theoretical explanation that the alliance is primarily a holistic experience for many clients. Bordin’s (1976, 1994) model of the working alliance implies that clients consider tasks, goals, and bond when assessing an alliance with a particular therapist. The holistic hypothesis, in contrast, suggests that alliance development can be rapid, implicit, and affect-based; clients cross a subjective threshold to experience the alliance as *established*. Similarly, Flückiger et al. (2018, p. 2) defined the alliance as “the holistic collaborative aspects of the therapist-client relationship.” They reported that the factor structure of WAI, California Psychotherapy Alliance Scale (CALPAS; Marmar et al., 1986), and Helping Alliance Questionnaire (HAQ; Alexander and Luborsky, 1987) share a common component of a “confident collaborative relationship” (2018, p. 3).

While this study provided further evidence of ceiling effects on working alliance and similar measures, future research should attempt to replicate consistent responses on alliance measures. Ceiling effects offer a potential marker of the holistic connection at the scale level, while consistent responding may provide a marker at the item level. Researchers may wish to conduct qualitative studies to investigate how individuals who provide consistent responses to alliance items perceive the alliance as well as how they interpret particular alliance items. In any event, distinguishing between social desirability and holistic hypotheses for high scores on alliance measures may be difficult in that these two constructs may produce similar effects. Finally, the use of archival data prevents hypothesis confirmation bias and effects from investigator expectancies, but also presents alternative explanations relative to choice of measures, varied timing of measurement administration, and participant selection and motivation. Four of the six measures in this study were homemade, for example, and timing of the administration of these measures may also have influenced observed results.

## Data availability statement

Publicly available datasets were analyzed in this study. This data can be found here: University of Michigan Inter-University Consortium for Political and Social Research (http://www.icpsr.umich.edu).

## Ethics statement

Ethical review and approval was not required for the study on human participants in accordance with the local legislation and institutional requirements. The patients/participants provided their written informed consent to participate in this study.

## Author contributions

The author confirms being the sole contributor of this work and has approved it for publication.

## Conflict of interest

The author declares that the research was conducted in the absence of any commercial or financial relationships that could be construed as a potential conflict of interest.

## Publisher’s note

All claims expressed in this article are solely those of the authors and do not necessarily represent those of their affiliated organizations, or those of the publisher, the editors and the reviewers. Any product that may be evaluated in this article, or claim that may be made by its manufacturer, is not guaranteed or endorsed by the publisher.

## Footnotes

1
http://www.icpsr.umich.edu
